# The effect of physiotherapists' explanation of therapeutic virtual reality on treatment expectations in healthy people and people with chronic musculoskeletal pain: Two online RCTs^☆^

**DOI:** 10.1016/j.pecinn.2025.100443

**Published:** 2025-11-17

**Authors:** Syl Slatman, Wim van Lankveld, J. Bart Staal, Harry van Goor, Raymond Ostelo, Janine Westendorp, Jesper Knoop

**Affiliations:** a Musculoskeletal Rehabilitation Research Group, HAN University of Applied Sciences, Nijmegen, the Netherlands; b Research Group Innovation of Human Movement Care, HU University of Applied Sciences, Utrecht, the Netherlands; c Radboud Institute for Health Sciences, Radboud University Medical Center, Nijmegen, the Netherlands; d Department of Surgery, Radboud University Medical Center, Nijmegen, the Netherlands; e Department of Health Sciences, Vrije Universiteit, Amsterdam, the Netherlands; f Department of Epidemiology and Data Science, Amsterdam UMC location Vrije Universiteit & Amsterdam Movement Sciences, Musculoskeletal Health, Amsterdam, the Netherlands; g Health, Medical, and Neuropsychology Unit, Leiden University, Leiden, the Netherlands

**Keywords:** Virtual reality (VR), Chronic musculoskeletal pain (CMP), Physiotherapy, Therapeutic communication, Randomized controlled trial

## Abstract

**Objectives:**

Chronic musculoskeletal pain (CMP) is a disabling condition, for which physiotherapy is a common treatment. Therapeutic virtual reality (VR) is an emerging treatment modality in physiotherapy care for patients with CMP. Treatment expectations of patients regarding therapeutic VR influence its effectiveness and could possibly be enhanced by the therapist by using positive language about therapeutic VR. The aim of the current studies was to explore the effect of physiotherapists explaining VR using positive versus neutral language, on treatment expectations of healthy participants (study 1) and patients with CMP (study 2).

**Methods:**

Two web-based, double-blinded RCTs were conducted with two groups (healthy participants and patients with CMP), that were randomly allocated to a video explaining therapeutic VR using positive language or neutral language. The primary outcome measures were treatment credibility and expectancy, assessed at baseline and post-intervention. Between-group differences and within-group changes were respectively analyzed using simple linear regression analyses and repeated measures ANOVAs. All analyses were performed separately for healthy participants and patients with CMP.

**Results:**

In total, 127 healthy participants (study 1) and 115 patients with CMP (study 2) were included and randomized. We found no between-group difference in treatment expectations between positive versus neutral language videos, neither in healthy participants nor patients with CMP. We found significant within-group changes for treatment expectations in both conditions (positive and neutral language) in healthy participants, and in the positive language condition only in the CMP group.

**Conclusions:**

The studies show that any explanation of therapeutic VR (both using positive or neutral language) seems to improve treatment expectations. Unexpectedly, using positive language was not superior to using neutral language. Future research should examine strategies for healthcare providers to set optimal treatment expectations on therapeutic VR in patients with CMP.

**Practice implications:**

A brief verbal explanation about therapeutic VR improves treatment expectations in both healthy participants and patients with CMP.

## Highlights

• Both positive and neutral language explanations of therapeutic VR appear to increase treatment expectations.
• Explaining therapeutic VR using positive language was not more effective than using neutral language.
• A short explanation by a physiotherapist on therapeutic VR may possibly improve treatment expectations.

## 1 Introduction

Chronic musculoskeletal pain (CMP), defined as pain lasting longer than three months, affects approximately 20 % of the adult population, leading to significant disability and burden in daily life [1]. CMP frequently impacts physical activities, social relations and occupation [2]. In general, treatment and management of CMP is challenging due to its complexity [3]. Pharmacological therapy often is ineffective [4] and can result in different side effects [5]. Non-pharmacological treatment options include among others, multidisciplinary rehabilitation, behavioral therapies (e.g. cognitive behavioral therapy (CBT)) and physiotherapy [6,7]. However, these treatments often only yield small and short-term results [8,9]. Recently, emerging technological treatment options were introduced as adjunct CMP treatments, including telehealth and virtual reality (VR) [10].

Therapeutic VR currently is in its infancy in physiotherapy care for patients with CMP and is mostly being used for patients with chronic neck pain, low back pain and generalized pain [11]. A recent meta-analysis showed that VR seems to be effective for patients with CMP [12]. However, patients with CMP generally lack knowledge about therapeutic VR [13], which could diminish the intervention's effectiveness and implementation. Mistrust in VR has been shown to be a barrier in implementing VR in healthcare, while patients feeling encouraged by their therapist has been shown to be a facilitator in implementing VR [14]. Expectations of a treatment influence the outcome [15,16], so it is important that physiotherapists explain the possibilities of therapeutic VR as a new treatment modality correctly and thoroughly in order to set right treatment expectations [17,18]. Previous studies showed that treatment expectations of patients can be altered by the communication style of therapists [19], which is presumed to be driven by placebo and nocebo effects [20]. Placebo (Latin for “I shall please”) and nocebo (Latin for “I shall harm”) effects represent complex and distinct phenomena, that are respectively defined as positive psychoneurobiological effects of the patient's positive expectation and negative psychoneurobiological effects of the patient's negative expectation [21]. Positive language use (e.g. “a success rate of 40%” instead of “a failure rate of 60%”) by the therapist could enhance the placebo effect [22,23]. Prior research estimated that in physiotherapy for patients with CMP, contextual factors like language use are responsible for at least 46 % of the overall treatment effect for exercise therapy and up to 87 % of the overall treatment effect for mobilizations [24]. Physiotherapists' verbal language use and the patients' expectations towards therapy are among the most important contextual factors [25]. However, the effect of physiotherapists' VR related positive or neutral language use on patients' treatment expectations has not been studied yet.

The aim of these studies was to explore the effect of physiotherapists' VR related positive or neutral language use on treatment expectations in both healthy participants (study 1) and patients with CMP (study 2). Our hypothesis was that the physiotherapist's VR explanation video with positive language use would be more effective than the video with neutral language on improving treatment expectations, in both studies. It should be noted that we did not aim to compare the results of this experiment between healthy participants and patients with CMP, as this would warrant age- and gender-matched controls.

## 2 Methods

### 2.1 Design

Two online, parallel, double-blinded RCTs, were conducted among Dutch participants and including healthy participants (study 1) and patients with CMP (study 2). The studies were conducted and reported following the Consolidated Standards of Reporting Trials (CONSORT) statement [26]. Randomization (1:1 ratio) and concealed allocation of participants was done by Qualtrics (Provo, Utah, USA). Data were collected between November 2023 and June 2024. Study 1 among healthy participants was initially conducted as a pilot to ensure feasibility and safety of the procedures. In addition, these data provide insights into the general mechanisms of treatment expectations and their perception on therapeutic VR. Both participants and researchers were blinded to intervention allocation. The two intervention videos were standardized in length, presenter, and setting, differing only in wording.

Approval was provided for each study separately by the ethical research committee of the HAN University of Applied Sciences (study 1: HAN ECO: 503.11/23) and (study 2: HAN ECO: 525.02/24). The studies were conducted in compliance with the Declaration of Helsinki [27]. Study 1 was registered retrospectively at ClinicalTrials.gov (identifier: NCT06307912) and study 2 was registered prospectively (identifier: NCT06282757). All participants signed online informed consent before data collection.

### 2.2 Participants

Participants for both studies were recruited online using convenience sampling through social media (and patient organizations in the CMP study) and were asked to participate in a study on their expectations of therapeutic VR. Recruitment of participants was done using a neutral message, requesting participants for research on expectations about therapeutic VR. Participants were eligible if they (1) were aged ≥18, (2) had access to the internet and (3) provided informed consent. They were excluded if they (1) lacked comprehension of the Dutch language and (2) had experience with therapeutic VR. Moreover, for study 2 with patients with CMP, participants were excluded if they did not suffer from CMP, while in study 1 with healthy respondents, participants were excluded if they suffered from CMP.

Sample size was determined a priori using G*Power 3.1 [28]. This analysis revealed a required total sample size of 102 participants per study (for both healthy participants and patients with CMP) to detect a medium effect (d = 0.5) with a power of 0.8 and one-sided 0.05 significance level. This effect size was based on the results of a comparable study regarding effects of an online video on low back pain education in people with low back pain [29]. With an expected drop-out of 10 %, we aimed to include 113 participants per study.

### 2.3 Interventions

**Fig. 1:**
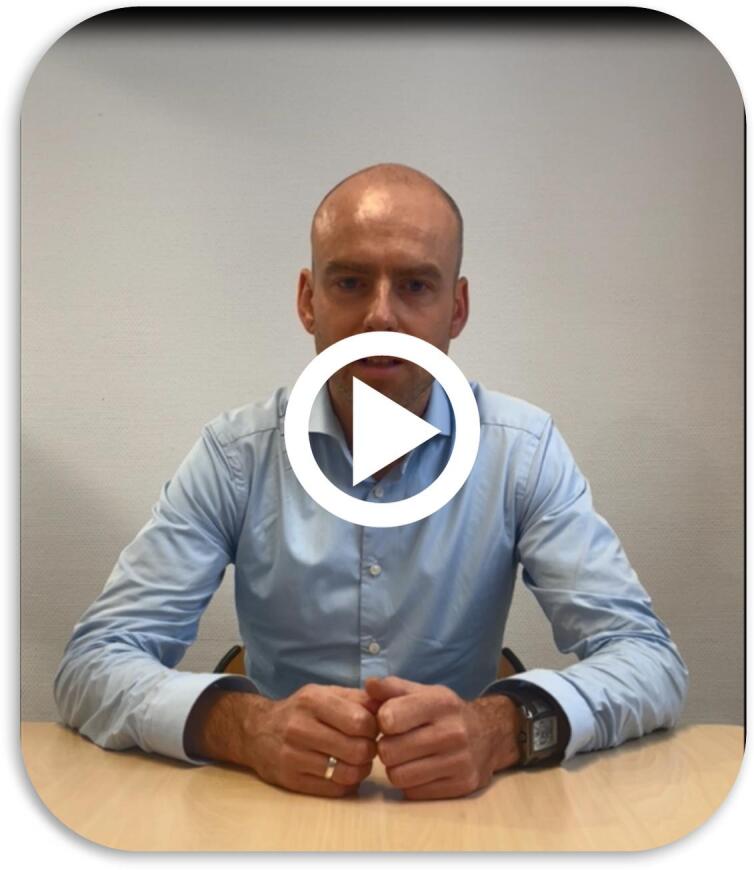
Still image from one of the VR explanation videos*.*

The development of the two explanatory scripts followed an iterative process combining evidence from literature and expert opinion. Content on the general principles of VR for chronic pain (e.g., immersion through 360° view and sound, and possible working mechanisms) was derived from prior research [11,30]. Evidence on therapeutic communication strategies (e.g., expectancy-enhancing text, and avoidance of nocebo-inducing phrasing) was informed by literature on placebo and nocebo communication [29,31]. Expert insights complemented the literature-based content, including input from an expert in therapeutic communication (WvL) and in placebo and nocebo communication (JW). One script included positive language, while the other included neutral language. Some general parts were similar in both scripts (e.g. “while using VR, you are immersed in a different world with 360° view and sound”), but the scripts differed on important aspects, as examples show in Table 1. These scripts were discussed and refined within the research group and with several VR and communication experts. The full scripts are added as appendix A. Finally, the scripts were transformed into two pre-recorded videos explaining VR therapy, as shown in Fig. 1. In both videos, participants saw a researcher (JK) addressing the camera directly, filmed in a neutral physiotherapy consultation setting with a plain background. Non-verbal communication (e.g., facial expression and eye contact) was similar in both videos to ensure comparability across conditions. Each video lasted approximately 1 min and 30 s. Prior to using them in the studies, both videos were pilot tested by 10 healthy participants using the same procedure as described in the next paragraph.

**Table 1:** Examples of positive and neutral language in intervention scripts.

Positive language	Neutral language
*“Virtual reality is a form of therapy that is increasingly being used.”*	*“Virtual reality is a relatively unknown technology that is used to a limited extent in physiotherapy practice, but increasingly in video games.”*
*“Promising results are found in previous research, which has shown that virtual reality can have a positive influence. Previous research showed that pain complaints decreased, patients became less anxious about their complaints and patients indicated that their quality of life improved.”*	*“Virtual reality shows effects on pain, frustrating symptoms such as mood, pain behavior, and also on quality of life. The virtual reality treatment has not yet been proven, but we can try it.”*
*“Your body may need some time to get used to using the VR headset, for example you may feel a bit disoriented. But fortunately the majority, namely 80 %, does not suffer from this.”*	*“You may feel a little dizzy or nauseous from the virtual environment. About 20 % of patients experience it as disorienting in the beginning”*

### 2.4 Procedure

**Fig. 2:**
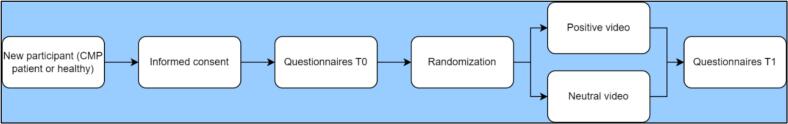
Study procedures*.*

Participants received a link to the online questionnaire in Qualtrics. After providing informed consent, participants were asked to answer the baseline questionnaires. Next, participants were randomized to either watch the positive or neutral language explanation video. Healthy participants of study 1 were asked to read and empathize with a case describing a common patient with CMP and respond accordingly, to help participants set their treatment expectations of therapeutic VR from a patient's perspective. Prior research showed that this is a reliable and valid approach for naïve participants to take on the role of patient [32]. Finally, all participants completed the post-intervention questionnaires. After completing these post-intervention questionnaires, participants were debriefed and informed about the purpose of the trial. Participants in the neutral language video arm also watched the positive language video, to get a more complete view of therapeutic VR. Fig. 2 provides an overview of the steps in the study procedure.

### 2.5 Measurements

#### 2.5.1 Demographics and potential confounders

The following demographic information was collected from all participants at baseline: age, gender and highest level of education, while only in study 2 with patients with CMP also pain duration and pain location were asked to report. Also at baseline, one question was included on digital skills (i.e. “how good are your computer skills”) on which patients scored themselves on a 1 (extremely bad) to 10 (extremely good) range, and one yes-no question was included on openness to therapeutic VR (i.e. “would you be open to treatment with therapeutic VR”).

#### 2.5.2 Outcomes

Before (at baseline) and after (post-intervention) the video, the Dutch version of the Credibility and Expectancy Questionnaire (CEQ) [33] was administered, as our primary outcome measure. The CEQ showed to be reliable and valid in measuring treatment credibility and expectancy [34]. This 11-item questionnaire was scored on a 9-point Likert scale and higher scores reflected better treatment credibility (possible range: 5–45) and expectancy (possible range: 6–54). Two subscales (i.e. attitude towards therapeutic VR and preference for therapeutic VR) of the Dutch version of the Attitudes Towards Virtual Reality Treatment (AVRT) [35] were administered at baseline and post-intervention, as secondary outcome measures. This questionnaire was only administered to patients with CMP, as the questionnaire was not yet publicly available when the study among healthy participants was conducted. The AVRT subscales included nine questionnaire items, that were scored on a 7-point Likert and higher scores reflected a more positive attitude towards VR treatment (possible range: 7–49) and preference for therapeutic VR (possible range: 2–14). The original English version of the AVRT showed sufficient reliability and validity [35], and was translated to Dutch for these studies by a bilingual (Dutch/English) speaker. The translated questionnaire was then checked by the research group and approved for use in these studies.

### 2.6 Analysis

The results of the studies were downloaded from Qualtrics and analyzed using SPSS version 27 (IBM, Armonk, USA). Continuous data was described as mean and standard deviation (SD), categorical data was described as frequency and percentage. The primary analysis was to test for between-group differences (positive vs. neutral language) on all treatment expectation outcome measures, using simple linear regressions. Baseline characteristics were included in the models as covariates. The secondary analysis was to test for within-group changes on all treatment expectation outcome measures using repeated measures ANOVAs. For both between-group differences and within-group changes, outcomes were reported as mean difference with 95 % confidence intervals (CIs). Statistical significance was set a *p* < .05. All analyses were performed separately for study 1 (healthy participants) and study 2 (patients with CMP).

## 3 Results

**Fig. 3:**
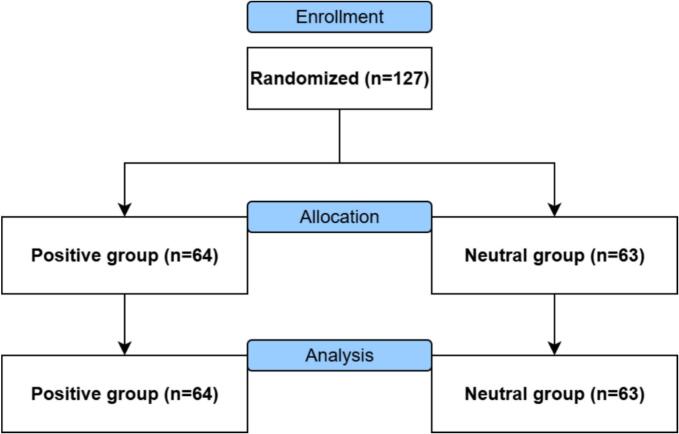
CONSORT diagram flow of healthy participants*.*

**Fig. 4:**
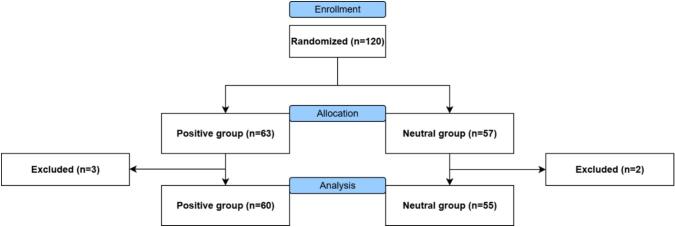
CONSORT diagram flow of patients with CMP*.*

In total, 247 participants completed the study procedures of this trial, of which 242 (98 %) were included for data-analysis, as shown in Fig. 3, Fig. 4. The five participants excluded did not have CMP (*n* = 4) or already received therapeutic VR (*n* = 1).

All participants received the positive or neutral language explanation video as allocated. More information on participant demographics is provided in Table 2. At baseline, no significant differences were found between the positive and neutral language explanation video arm of healthy participants in study 1 and positive and neutral language explanation video arm of patients with CMP in study 2.

**Table 2:** Baseline characteristics*.*

	Study 1: Healthy participants (*n* = 127)		Study 2: Patients with CMP (*n* = 115)	
	Positive video (*n* = 64)	Neutral video (*n* = 63)	Positive video (n = 60)	Neutral video (*n* = 55)
Age, n (%)				
18–30	31 (48)	23 (37)	11 (18)	12 (22)
31–40	6 (9)	4 (6)	3 (5)	3 (5)
41–50	2 (3)	10 (16)	6 (10)	14 (25)
51–60	9 (14)	13 (21)	11 (18)	12 (22)
60+	16 (25)	13 (21)	29 (48)	14 (25)
Gender, female, n (%)	33 (52)	42 (67)	53 (88)	47 (86)
Education level, n (%)				
Lower	10 (16)	10 (16)	11 (18)	6 (11)
Middle	46 (72)	39 (62)	38 (63)	41 (75)
Higher	8 (13)	14 (22)	11 (18)	8 (15)
Computer skills, mean (SD)	7.6 (1.3)	7.7 (1.0)	7.4 (1.3)	7.7 (1.5)
Open to VR, n (%)	57 (89)	60 (95)	52 (87)	49 (89)
Pain duration, n (%)	n/a	n/a		
3–6 months			6 (10)	4 (7)
7–12 months			7 (12)	5 (9)
1–5 years			18 (30)	15 (27)
6–10 years			10 (17)	15 (27)
11–20 years			14 (23)	13 (24)
20+ years			5 (8)	3 (5)
Pain location, n (%)	n/a	n/a		
Generalized pain			20 (33)	23 (42)
Cervical pain			3 (5)	4 (7)
Lumbar pain			23 (38)	12 (22)
Upper extremity pain			4 (7)	6 (11)
Lower extremity pain			10 (17)	10 (18)

CMP = chronic musculoskeletal pain, n/a = not available, SD = standard deviation, VR = virtual reality.

### 3.1 Between-group differences

No statistical significant between-group differences (positive vs. neutral language) were found for any of the outcome measures, both in study 1 (healthy participants) and study 2 (patients with CMP). More specifically, in both healthy participants, treatment credibility (mean difference (MD): -1.6, 95 % CI: −4.9 − 1.6) and expectancy (MD: −0.7, 95 % CI: −4.9-3.4) and in patients with CMP, treatment credibility (MD: -1.6, 95 % CI: −5.1-1.9) and expectancy (MD: -1.3, 95 % CI: −5.7-3.2) showed no significant differences post-intervention. See Table 3 for all primary outcome measures within- and between-group results. In patients with CMP, no significant between-group differences were found on the AVRT questionnaire (Table 4).

**Table 3:** Within- and between-group results of primary outcome measures*.*

		Study 1: Healthy participants			Study 2: Patients with CMP		
		Positive video (n = 64)	Neutral video (n = 63)	Between-group difference	Positive video (n = 60)	Neutral video (n = 55)	Between-group difference
Treatment credibility, mean (SD)	Baseline	23.0 (9.5)	23.2 (13.0)		21.1 (7.8)	21.4 (9.8)	
	Post-intervention	28.4 (9.9)	26.7 (8.6)		24.2 (8.8)	22.6 (9.9)	
	Mean (within-group) change (95 %CI)	5.4 (3.8–6.9)	3.5 (1.8–5.3)		3.1 (1.7–4.5)	1.2 (−0.5–2.8)	
	Mean (between-group) difference (95 %CI)			-1.6 (−4.9–1.6)			-1.6 (−5.1–1.9)
	P	<0.001⁎	<0.001⁎	0.206	<0.001⁎	0.163	0.371
Treatment expectancy, mean (SD)	Baseline	27.6 (12.1)	29.8 (11.5)		25.2 (10.7)	25.1 (11.6)	
	Post-intervention	32.9 (12.1)	32.5 (11.3)		27.2 (11.7)	26.0 (12.3)	
	Mean (within-group) change (95 %CI)	5.4 (3.6–7.1)	2.7 (0.5–5.0)		2.1 (0.4–3.7)	0.9 (−1.1–2.9)	
	Mean (between-group) difference (95 %CI)			−0.7 (−4.9–3.4)			−1.3 (−5.7–3.2)
	P	<0.001⁎	0.018⁎	0.271	0.018⁎	0.382	0.450

CI = confidence interval, CMP = chronic musculoskeletal pain, SD = standard deviation, VR = virtual reality.

⁎ Significant for *p* < .05.

**Table 4:** Within- and between-group results of secondary outcome measures in patients with CMP.

		Positive video (*n* = 60)	Neutral video (n = 55)	Between-group difference
Attitude towards therapeutic VR, mean (SD)	Baseline	5.0 (0.9)	5.1 (0.9)	
	Post-intervention	5.2 (1.0)	5.1 (1.0)	
	Mean (within-group) change (95 %CI)	0.1 (−0.1–0.3)	−0.1 (−0.3–0.1)	
	Mean (between-group) difference (95 %CI)			−0.1 (−0.5–0.2)
	P	0.168	0.387	0.356
Preference for VR, mean (SD)	Baseline	3.3 (1.3)	3.2 (1.5)	
	Post-intervention	3.5 (1.3)	3.3 (1.4)	
	Mean (within-group) change (95 %CI)	0.2 (−0.0–0.4)	0.2 (−0.1–0.5)	
	Mean (between-group) difference (95 %CI)			−0.1 (−0.7–0.4)
	P	0.067	0.171	0.336

CI = confidence interval, CMP = chronic musculoskeletal pain, SD = standard deviation, VR = virtual reality.

### 3.2 Within-group changes

In study 1 (healthy participants) within-group changes were found for both treatment credibility and expectancy, for both the positive and neutral language explanation video. Within-group analyses of study 2 (patients with CMP) showed that the positive language explanation video improved both treatment credibility and expectancy, while the neutral language explanation video did not change either of these outcomes. However, no statistically significant changes were found for attitude towards VR and preference for VR in the positive and neutral language explanation arm of patients. Moreover, in patients with CMP, no significant within-group changes were found for the AVRT questionnaire.

## 4 Discussion and conclusion

### 4.1 Discussion

These studies aimed to explore the effect on VR treatment expectations of physiotherapists' therapeutic VR explanation using positive or neutral language in both healthy participants and patients with CMP. We found no between-group differences between the positive and neutral language videos on treatment expectations, neither for healthy participants and patients with CMP. On the other hand, we did find within-group changes in treatment expectations for both the positive and neutral language in healthy participants and for the positive language only in patients with CMP. Post hoc power to detect the observed effect (d = 0.17) was 23 %, indicating a 77 % probability of a Type II error [36]. Due to the low power, our sample size was likely insufficient to detect the smaller-than-anticipated effect, and conclusions regarding the absence of effect should be interpreted with caution.

Our finding that the positive language explanation video was not more effective in improving treatment expectations than the neutral language explanation video was not expected. Prior studies showed that positive language generally works better than neutral language in physiotherapy, partly due to the placebo effect [20,37]. There are several possible explanations for this unexpected finding in our study, besides the possibility of an underpowered trial. First of all, therapeutic VR is still a novel technology and relatively unknown among most people [13]. All new information about this technology might therefore improve treatment expectations, as treatment understanding can improve treatment expectations [15,38]. Furthermore, it is possible that the wording of the neutral explanation video was not perceived as neutral about VR by all participants, but also as a positive explanation about VR. Future studies could test this using for example a manipulation check [39] and should include sufficiently contrasting explanation videos. Finally, it can be hypothesized that neutral communication about VR might be perceived as an indication of a knowledgeable physiotherapist, and therefore improve treatment expectations [40].

Another finding of these studies is that both healthy participants and patients with CMP seemed to have a positive attitude towards therapeutic VR, as for example in both groups around 90 % of the participants were open to using VR at baseline. This is congruent with the results of a recent survey on the acceptability of therapeutic VR among patients with CMP [41]. However, prior research showed that a therapist's perceived lack of open-minded patients is a barrier in VR implementation in healthcare [14]. This implies that it could be possible that physiotherapists hold misbeliefs about patients with CMP's openness to using new technological interventions like VR.

The main strength of these studies was including both healthy participants and patients with CMP, which enabled us to learn more about participants' perception on therapeutic VR, as this knowledge is limited for both healthy participants [35] and patients with CMP [13].

The following limitations should be noted for these studies. First, the baseline questionnaires, intervention and post-test questionnaires were administered within the same session. This could have led to social-desirability bias as participants might remember their initial responses. Second, these studies did not mimic the natural physiotherapy setting, since the videos were shown online and participants for example were not able to ask questions regarding the intervention. Third, these studies did not include any follow-up measurements, which lacked the possibility to study longer-term effects of the videos. Fourth, participants were recruited with a text inviting them to a study on their expectations about therapeutic VR. This could have induced a sampling bias with participants that are more positive about therapeutic VR. Finally, this study did not include a no-intervention control group, so it is unclear whether the observed study results were solely due to the intervention or for example to participants' a priori expectation of using VR. Without a no-intervention control condition, these potential effects cannot be ruled out and interpretation of results should be done with caution.

Future studies should study treatment expectations for therapeutic VR in more detail, as much is still unknown about the context when administering digital therapeutics like VR [42]. These studies should examine the best communication strategies physiotherapists could employ to set the optimal therapeutic VR treatment expectations in patients with CMP. To better reflect clinical physiotherapy practice, they could for example include the possibility for patients to express doubts and ask questions, while also keeping non-verbal communication in mind [43] and include a try-out for patients, as a prior study showed that acceptance and attitudes towards VR improved in healthy participants after testing VR [44]. Future studies are needed into real-world physiotherapy practices to understand how therapeutic VR is currently introduced and explained to patients. Qualitative studies could be incorporated to explore how patients perceive different communication strategies and whether these perceptions influence their motivation, engagement, or expectations regarding therapeutic VR [45]. These studies should incorporate follow-up measurements to study longer-term effects of altering treatment expectations. Finally, they could examine to what extent the results of this study are applicable to other emerging forms of digital health in CMP, like videoconferencing and mHealth [10].

### 4.2 Practice implications

In spite of these limitations, these studies have several important implications. First of all, the studies underline the importance of information. A brief verbal explanation of a new therapeutic technology seems to alter treatment expectations in both healthy participants and patients with CMP. The effect of the positive language explanation video was similar to that of the neutral language explanation video, which underlines the importance of providing information on the intervention to modify and improve treatment expectations [46,47]. In providing this information, it seems that using both neutral or positive language is acceptable, while prior research showed that negative language use should preferably be avoided [29,48]. This will help patients with CMP, as treatment expectations have been shown to influence treatment satisfaction and clinical outcome measures [49,50]. Although it should also be stated that unrealistic treatment expectations might diminish treatment effects [51]. Therefore, it is important to use communication that raises positive but realistic expectations. To aid physiotherapists in doing this, it might be valuable to offer them communication training to improve treatment expectations [29,52].

## 5 Conclusion

In conclusion, we found that effects on treatment expectations did not seem to differ between a physiotherapist's short explanation on therapeutic VR as a treatment modality for CMP using positive language versus neutral language. This was found consistently in both healthy participants and patients with CMP. Future research should examine physiotherapists' communication strategies, to optimally set CMP patients' treatment expectations regarding therapeutic VR.

## Authors contributions

SS was the principal investigator of these studies and drafted the first version of the manuscript. WvL conceptualized and designed these studies and reviewed and revised the final manuscript. JBS reviewed and revised the final manuscript and supervised SS. HG reviewed and revised the final manuscript and supervised SS. RO reviewed and revised the final manuscript and supervised SS. JW conceptualized the studies and reviewed and revised the final manuscript. JK conceptualized and designed the studies, reviewed and revised the manuscript and supervised SS.

## CRediT authorship contribution statement

**Syl Slatman:** Writing – original draft, Project administration, Methodology, Investigation, Formal analysis, Data curation, Conceptualization. **Wim van Lankveld:** Writing – review & editing, Supervision, Conceptualization. **J. Bart Staal:** Writing – review & editing, Supervision, Methodology. **Harry van Goor:** Writing – review & editing, Supervision. **Raymond Ostelo:** Writing – review & editing, Supervision. **Janine Westendorp:** Writing – review & editing, Investigation, Conceptualization. **Jesper Knoop:** Writing – review & editing, Supervision, Methodology, Conceptualization.

## Consent for publication

Not applicable.

## Ethics approval and consent to participate

These studies were conducted according to the Declaration of Helsinki. Ethical approval for these studies was obtained from the ethics committee of the HAN University of Applied Sciences (case numbers: 503.11/23 and 525.02/24). Participants provided informed consent before answering the survey.

## Funding

These studies were funded by 10.13039/501100001826ZonMw (case number: 10270032021502). The funder had no role in the design, organization and execution of the study.

## Declaration of competing interest

The authors declare they have no competing interests.

## Data Availability

The data generated during these studies will not be publicly available, but will be available upon reasonable request to the corresponding author.

## Appendix A Script texts

### A.1 Positive explanation (original in Dutch)

Virtual reality is een therapievorm, die in toenemende mate wordt toegepast. Tijdens het gebruik van een virtual reality bril wordt u ondergedompeld in een andere wereld met 360° beeld en geluid. In deze omgeving, waarin de echte wereld is nagemaakt, kunt u informatie krijgen over mogelijke technieken die uw pijn kunnen verminderen. U kunt bijvoorbeeld oefenen met bewegen in spelvormen die uw klachten kunnen doen afnemen, of u kunt een programma kiezen waarin u leert om uw aandacht te verleggen en minder de focus te hebben op uw pijnklachten.

Het voordeel van virtual reality is dat de behandeling kan worden gepersonaliseerd aan uw eigen behoefte. U kan het thuis gebruiken wanneer u wilt. Daarnaast vormt virtual reality een andere wereld met meerdere vormen van feedback, zoals geluid en beeld. Wetenschappelijk onderzoek laat zien dat aan de hand van deze feedback, virtual reality verschillende netwerken in de hersenen activeert. Deze activatie versterkt het leren en het herstel van de klachten. Er worden veelbelovende resultaten gevonden bij eerder gedaan onderzoek, waarbij is aangetoond dat virtual reality een positieve invloed kan hebben. Eerder onderzoek liet zien dat pijnklachten afnamen, patiënten minder angstig werden over hun klachten en de patiënten gaven aan dat hun kwaliteit van leven verbeterde. Het kan zijn dat uw lichaam even moet wennen aan het gebruiken van een VR bril, u kunt u bijvoorbeeld wat gedesoriënteerd voelen. Maar gelukkig heeft het merendeel, namelijk 80 %, hier geen last van. Daarnaast hebben voorgaande patiënten ook aangegeven de therapie beter te kunnen volhouden als er gebruik wordt gemaakt van virtual reality-therapie.

### A.2 Positive explanation (translated to English)

Virtual reality is a form of therapy that is increasingly being used. When using a virtual reality headset you are immersed in another world with 360° image and sound. In this environment, which recreates the real world, you can learn about possible techniques that can reduce your pain. For example, you can practice exercising in game forms that can reduce your complaints, or you can choose a program in which you learn to shift your attention and focus less on your pain complaints.

The advantage of virtual reality is that the treatment can be personalized to your own needs. You can use it at home whenever you want. In addition, virtual reality is a different world with multiple forms of feedback, like sounds and images. Scientific research shows that based on this feedback, virtual reality activates different networks in the brain. This activation enhances learning and recovery from complaints. Promising results are found in previous research, which has shown that virtual reality can have a positive influence. Previous research showed that pain complaints decreased, patients became less anxious about their complaints and patients indicated that their quality of life improved. Your body may need some time to get used to using the VR headset, for example you may feel a bit disoriented. But fortunately the majority, namely 80 %, does not suffer from this. In addition, previous patients have also indicated that they are able to maintain therapy better if virtual reality therapy is used.

### A.3 Neutral explanation (original in Dutch)

Virtual reality is een relatief onbekende technologie die nog beperkt wordt toegepast in de fysiotherapiepraktijk, maar al meer bij videogames. Tijdens het gebruik van een virtual reality bril wordt u ondergedompeld in een andere wereld door 360° beeld en geluid. In deze omgeving, waarin de echte wereld is nagemaakt, kunt u informatie krijgen over de pijn die u ervaart, u kunt aan de slag gaan met bewegen in spelvormen of u kunt een programma kiezen ter afleiding van de pijn.

De virtual reality behandeling kan worden gepersonaliseerd aan uw persoonlijke behoefte. Daarnaast vormt virtual reality een omgeving met meerdere vormen van prikkels, zoals geluid en beeld. Aan de hand van deze prikkels kan virtual reality verschillende netwerken in de hersenen activeren. Deze prikkeling versterkt het leren en het herstel van de pijn die u ervaart. Naast afleiding is onbekend op welke werkingsmechanismen het nog meer effect heeft. Virtual reality laat effecten zien op pijn, frustrerende symptomen zoals gemoedstoestand, pijngedrag, en ook op de kwaliteit van leven. De virtual reality behandeling is nog niet bewezen, maar we kunnen het proberen. Het is echter wel mogelijk dat u wat duizelig of misselijk wordt van de virtuele omgeving. Door ongeveer 20 % van de patiënten wordt dit in het begin als desoriënterend ervaren en ik heb hier zelf eigenlijk ook wel eens last van.

### A.4 Neutral explanation (translated to English)

Virtual reality is a relatively unknown technology that is used to a limited extent in physiotherapy practice, but increasingly in video games. When using a virtual reality headset you are immersed in another world with 360° image and sound. In this environment, which recreates the real world, you can receive information about the pain you experience, you can start exercising using games or you can choose a program to distract from the pain.

The virtual reality treatment can be personalized to your personal needs. In addition, virtual reality provides an environment with multiple forms of stimuli, such as sounds and images. Based on these stimuli, virtual reality can activate different networks in the brain. This stimulation enhances learning and recovery from the pain you experience. In addition to distraction, it is unknown which other working mechanisms it has an effect on. Virtual reality shows effects on pain, frustrating symptoms such as mood, pain behavior, and also on quality of life. The virtual reality treatment has not yet been proven, but we can try it. However, you may feel a little dizzy or nauseous from the virtual environment. About 20 % of patients experience it as disorienting in the beginning and I actually suffer from this myself sometimes.
